# Evaluation of Oxidative Status in Elderly Patients with Multiple Cerebral Infarctions and Multiple Chronic Total Coronary Occlusions

**DOI:** 10.1155/2022/2083990

**Published:** 2022-06-28

**Authors:** Xia Li, Dianxuan Guo, Youdong Hu, Ying Chen

**Affiliations:** Xiamen Road Branch Hospital, The Affiliated Huaian Hospital of Xuzhou Medical University, Huaian 223002, China

## Abstract

**Background:**

Oxidative stress plays a key role in atherosclerosis. Acting via high level of reactive oxygen species, an increase of oxidative stress is involved in the pathogenesis and progression of atherosclerostic stenosis or occlusion of arteries. Oxidative stress leads to an accumulation of oxidized low-density lipoprotein, which plays important roles in steno-occlusion of cerebral and coronary arteries. However, the exact reasons for multiple cerebral and coronary artery steno-occlusion in elderly patients remain unclear. The aim was to evaluate the effects of imbalance of oxidative/antioxidative status on concomitant multiple brain infarcts and multiple chronic total coronary occlusions in elderly patients.

**Methods:**

We measured the circulating levels of malondialdehyde (MDA), reactive oxygen species (ROS), thiobarbituric acid reactive substance (TBARS), advanced oxidation protein products (AOPP), superoxide dismutase 1 (SOD 1), superoxide dismutase 2 (SOD 2), superoxide dismutase 3 (SOD 3), and paraoxonase 1 (PON 1) in patients with concomitant multiple cerebral infarcts and multiple chronic total coronary occlusions.

**Results:**

Circulating levels of oxidative stress markers (MDA, ROS, TBARS, and AOPP) were increased (*P* < 0.001) and antioxidative stress markers (SOD 1, SOD 2, SOD 3, and PON 1) were decreased (*P* < 0.001) in elderly patients with concomitant multiple brain infarcts and multiple chronic total coronary occlusions.

**Conclusions:**

The findings suggested that the imbalance of oxidative/antioxidative status may be associated with multiple cerebral infarcts and multiple chronic total coronary occlusions and may contribute to the development of concomitant multiple brain infarcts and multiple chronic total coronary occlusions in elderly patients.

## 1. Introduction

The vascular endothelial dysfunctions are involved in the pathophysiology of vascular diseases and play an important role in the development of artery atherosclerotic occlusive diseases [1]. The vascular endothelial cell injury and dysfunction are related to oxidative stress and contribute to the development and progression of artery atherosclerotic occlusive diseases [1].

Malondialdehyde- (MDA-) modified low-density lipoprotein is considered the risk markers of oxidative stress and cardiovascular diseases. A reduction of MDA-modified low-density lipoprotein is considered to be beneficial for inhibiting the development of oxidative stress [2]. Oxidative stress is promoted by abnormal generation of reactive oxygen species (ROS), and ROS generation is considered as a key mechanism in atherosclerosis [3]. The overproduction of ROS is involved in arterial injury and leads to the prooxidant/antioxidant imbalance and plays an important role in the progress of atherosclerosis [3]. There are ongoing studies to identify new therapeutic methods to selectively target oxidative stress response in atherosclerosis [3]. Thiobarbituric acid reactive substance (TBARS) is the markers of oxidative stress and atherosclerosis, and it increases endothelial cell cytotoxicity [4] and is considered as a positive determinant of atherosclerosis. Oxidative stress is increased by TBARS and leads to carotid artery intima-media thickness used as indicator of atherosclerosis [5]. Advanced oxidation protein products (AOPP) as the biomarker of oxidative stress induce ROS generation, and oxidative stress is a consequence of the imbalance of the production and removal of ROS. AOPP is an indicator of oxidant-induced protein damage and plays the key roles in the damages of cells and organs and pathological process of diseases [6]. Oxidative damage leads significantly to the progression of atherosclerosis, and superoxide dismutase 1 (SOD 1) reduces the expressions of oxidative stress-related genes and the risk of atherosclerosis [7]. Superoxide dismutase 2 (SOD 2) suppresses atherosclerotic plaques and atherosclerotic development. Oxidized low-density lipoprotein is increased in patients with atherosclerosis, and oxidized low-density lipoprotein promotes ROS production and leads to the progression of atherosclerosis through inhibiting SOD 2 expression [8]. Superoxide dismutase 3 (SOD 3) as an antioxidative microsomal enzyme is highly expressed in arterial walls where it decreases oxidative stress response. SOD 3 also plays a key role in decreasing atherosclerotic risk and ROS-mediated inflammatory response [9]. Paraoxonase 1 (PON 1) as a cellular protector against oxidative damages inhibits low-density lipoprotein oxidation and plays the important roles in reducing the oxidative stress, atherosclerosis, and risk for cardiac diseases through removal of low-density lipoprotein oxidation [10].

Because there is no clear evidence for association of oxidative/antioxidative status and concomitant multiple cerebral infarcts and multiple coronary chronic total occlusions in elderly patients, the aim of the study was to assess markers of the oxidative stress and antioxidative stress (concentrations of MDA, ROS, TBARS, AOPP, SOD 1, SOD 2, SOD 3, and PON 1) and determine the association of oxidative/antioxidative status and concomitant multiple cerebral infarcts and multiple chronic total coronary occlusions in elderly patients.

## 2. Materials and Methods

### 2.1. Patient Selection

From 1 January 2010 to 31 December 2018, our research included the patients with multiple cerebral infarcts (MCI) and multiple chronic total coronary occlusions in different groups. The inclusion criteria adopted in this research were (1) the patients aged 55 to 87 years and (2) the patients with MCI defined as brain infarcts in multiple artery territories+one-vessel coronary lesion (OVCL) [11], MCI+two-vessel coronary lesion (TVCL), and MCI+multiple-vessel coronary lesion (MVCL). The research was approved by the Xuzhou Medical University and Human Research Ethics Committee of the University according to the relevant Chinese laws, and the written informed consents were obtained from human participants in the research according to the principles in the Revised Declaration of Helsinki. The patients with one or more of the following criteria were excluded from the research: (1) acute cerebral infarctions, (2) acute myocardial infarctions, (3) chronic artery occlusions of the upper limb, (4) chronic artery occlusion of the lower extremity, (5) cancers, and (6) using antioxidative medicines.

### 2.2. Research Protocol

The healthy participants were included in control (CON) group (*n* = 61). The numbers of patients with MCI+OVCL, MCI+TVCL, and MCI+MVCL were 230, 222, and 213, respectively. The patients with MCI+chronic total coronary occlusions defined as 100% obstruction of a coronary artery [12] were included in MCI+one-vessel coronary occlusion (OVCO) group (*n* = 59), MCI+two-vessel coronary occlusion (TVCO) group (*n* = 57), MCI+multiple-vessel coronary occlusion (MVCO) defined as three-vessel coronary occlusions [13] group (*n* = 54), unilateral multiple cerebral infarcts (UMCI)+OVCO group (*n* = 57), bilateral multiple cerebral infarcts (BMCI)+TVCO group (*n* = 55), BMCI+MVCO group (*n* = 53), multiple lacunar cerebral infarcts (MLCI)+OVCO group (*n* = 57), multiple cerebellum infarcts (MCBI)+TVCO group (*n* = 55), MCBI+MVCO group (*n* = 53), multiple cerebral microinfarcts (MCMI) (≤4 mm)+OVCO group (*n* = 57), multiple small cerebral infarcts (MSCI) (diameter < 15 mm)+TVCO group (*n* = 55), and multiple large cerebral infarcts (MLCI) (diameter ≥ 15 mm)+MVCO group (*n* = 53).

### 2.3. Evaluations of Cerebral Infarcts and Coronary Occlusions

Magnetic resonance imaging (Philips 3.0 T scanner) was used for MCI. The scanner was done with 6 mm slice thickness and interslice 1 mm. The imaging data were used for evaluating MCI. The imaging data were determined by three experienced imaging specialists [14]. The coronary occlusion evaluations in quantitative coronary angiography were measured by a digital subtraction angiographic method. Coronary angiography and coronary occlusions were evaluated by three trained interventional cardiologists blinded to the diagnoses of the patients [15].

### 2.4. Evaluations of MDA, ROS, TBARS, and AOPP

Fasting plasma samples were used for MDA, and MDA levels were determined by thiobarbituric acid reacting substance assays using the spectrophotometer at 534 nm wavelength [16]. ROS was evaluated by flow cytometer (FACSCalibur cytometer, Becton Dickenson, San Jose, CA, USA). For each evaluation, 10^4^ events were collected and the clinical data were assessed using the CellQuest software. The plasma ROS levels were expressed as mean fluorescence intensity (MFI) [17]. The venous blood samples were collected by tubes with heparin and then were centrifuged at 3,000 rpm for 35 min at 4°C. The plasma blood samples were stored at -70°C until further processing, and TBARS levels were assessed fluorometrically by the TBARS assay kit (the Oxitek TBARS Assay Kit, Zepto Metrix Corporation, Buffalo, New York) according to the instruction of the kit [18]. AOPP evaluations were based on spectrophotometric assays, fasting plasma samples (200 *μ*L) were diluted 1/5 in sterile phosphate buffer, and 200 *μ*L of chloramine T was used for measuring curve. The plasma samples were loaded into a microtiter plate, and final absorbances were determined at 340 nm by a spectrophotometer. The AOPP levels were expressed as *μ*mol/L [19].

### 2.5. Measurements of SOD 1, SOD 2, SOD 3, and PON 1

SOD 1 plasma levels were determined using the SOD 1 enzyme-linked immunosorbent assay kit, and the blood samples were incubated for 60 min at the room temperature with anti-SOD 1 antibody. The colorimetric substrates were added for 10 min and the reactions were stopped and all plates were read at 450 nm [20]. The blood plasma SOD 2 was assessed by SOD 2 assay kit with a tetrazolium salt solution, and tetrazolium salt assay was used for detecting the superoxide radicals. Both SOD 1 and SOD 3 were inhibited for detecting SOD 2, and SOD 2 activity was measured by determining the absorbance at 440 nm [21]. The blood plasma activities of SOD 3 were assayed based on the SOD 3 assay kit according to the manufacturer's instructions. The microtiter plate wells were coated with a specific capture antibody for targeting antigen and the plate wells were read at 450 mm [21]. The activities of plasma PON-1 were measured using the manufacturer's instructions. The PON-1 hydrolysis product of paraoxon released p-nitrophenol, which rate of formation was measured by spectrophotometrically with recording absorbances at 405 nm [22].

### 2.6. Statistical Analysis

The results of clinical data on the biomarker levels of oxidative stress and antioxidative stress were presented as the mean plus or minus standard deviations (mean ± SD). The normality of the variables was checked using the Shapiro-Wilks test to determine whether the variables fit a normal distribution. All *P* values from the Shapiro-Wilks tests were greater than 0.05, which indicated all variables were normally distributed. The chi-square test was used to compare categorical data. The one-way analysis of variance (ANOVA) was performed to determine the variances in the different groups. Multiple regression statistical analysis was employed to examine all independent risk factors for the MCI and multiple chronic total coronary occlusions (MCTCO). The statistical and clinical significances in all research groups were considered when *P* values were less than 0.05. The statistical analyses of the participant's clinical data were entered and analyzed using statistical package for the SPSS (24.0) software programs for conducting the statistical analysis (SPSS, IBM Corporation, Armonk, New York, USA) in evaluating different levels of MDA, ROS, TBARS, AOPP, SOD 1, SOD 2, SOD 3, and PON 1.

## 3. Results

### 3.1. The Participant Baseline Characteristics in the Study

The characteristics of participants were very similar among different research groups (Table 1). All participants in different research groups were well-matched without any statistically significant differences in gender and age.

Definitions of our research as follows: (1) coronary artery disease (CAD) is defined as stenosis of more than 50% of coronary artery [23]. (2) Transient ischemic attack (TIA) is defined as a transient episode of neurologic dysfunction resulting from regional cerebral ischemia [24]. (3) Family histories of stroke (FHOS) is defined as patients with a parental histories of stroke [25]. (4) Hypertension (HT) is defined as an arterial blood pressure greater than 140/90 mmHg [26]. (5) Diabetes mellitus (DM) diabetes is defined as fasting plasma glucose of more than 7.0 mmol/L [27]. (6) Peripheral vascular disease (PVD) is defined as the ankle-brachial blood pressure index < 0.90 [28]. (7) Smoking is defined as smoking > 20 cigarettes per day [29]. (8) Alcohol consumption (AC) is defined as a score of ≥6 for women or ≥8 for men on the Alcohol Use Disorders Identification Test [30]. (9) Myocardial infarction (MI) is defined as sudden cardiac ischemic death of myocardial tissue [31]. (10) Angina pectoris (AP) is defined as the pain and discomfort in the area of the chest [32]. (11) Duration of illness (DOI) is defined as the number of years since the first hospitalization [33].

### 3.2. Levels of MDA, ROS, TBARS, AOPP, SOD 1, SOD 2, SOD 3, and PON 1 in Patients with MCI+OVCO, MCI+TVCO, and MCI+MVCO

The levels of MDA, ROS, TBARS, and AOPP were increased in MCI+TVCO group when compared with CON and MCI+OVCO groups, respectively (*P* < 0.001), and were further increased in MCI+MVCO group compared to MCI+OVCO and MCI+TVCO groups, respectively (*P* < 0.001). The expression levels of SOD 1, SOD 2, SOD 3, and PON 1 were decreased in MCI+TVCO group when compared with CON and MCI+OVCO groups, respectively (*P* < 0.001) and were further decreased in MCI+MVCO group compared to MCI+OVCO and MCI+TVCO groups, respectively (*P* < 0.001). Changes of MDA, ROS, TBARS, AOPP, SOD 1, SOD 2, SOD 3, and PON 1 indicated an increase in the severity of concomitant brain infarcts and coronary occlusions in patients. The results proved that there was a significant relationship between levels of prooxidative stress/antioxidative stress markers and concomitant MCI and coronary chronic total occlusion in patients (Table 2).

### 3.3. Levels of Prooxidative Stress Markers/Antioxidative Stress Markers in Patients with UMCI+OVCO, BMCI+TVCO, and BMCI+MVCO

The levels of MDA, ROS, TBARS, and AOPP were increased in BMCI+TVCO group when compared with CON and UMCI+OVCO groups, respectively (*P* < 0.001), and were further increased in BMCI+MVCO group compared to UMCI+OVCO and BMCI+TVCO groups, respectively (*P* < 0.001). The expression levels of SOD 1, SOD 2, SOD 3, and PON 1 were decreased in BMCI+TVCO group when compared with CON and UMCI+OVCO groups, respectively (*P* < 0.001), and were further decreased in BMCI+MVCO group compared to UMCI+OVCO and BMCI+TVCO groups, respectively (*P* < 0.001). The results suggested that the prooxidative stress/antioxidative stress markers could be used as independent risk factors for the prediction of UMCI+OVCO, BMCI+TVCO, and BMCI+MVCO in patients, and these eight markers were proposed to evaluate the diagnostic possibility of UMCI+OVCO, BMCI+TVCO, and BMCI+MVCO in patients (Table 3).

### 3.4. Biomarker Levels in Patients with MLCI+OVCO, MCBI+TVCO, and MCBI+MVCO

The levels of MDA, ROS, TBARS, and AOPP were increased in MCBI+TVCO group when compared with CON and MLCI+OVCO groups, respectively (*P* < 0.001), and were further increased in MCBI+MVCO group compared to MLCI+OVCO and MCBI+TVCO groups, respectively (*P* < 0.001). The expression levels of SOD 1, SOD 2, SOD 3, and PON 1 were decreased in MCBI+TVCO group when compared with CON and MLCI+OVCO groups, respectively (*P* < 0.001), and were further decreased in MCBI+MVCO group compared to MLCI+OVCO and MCBI+TVCO groups, respectively (*P* < 0.001). As shown in Table 4, the high levels of MDA, ROS, TBARS, and AOPP through inhibiting the concentrations SOD 1, SOD 2, SOD 3, and PON 1 promoted the development of MCI+coronary chronic total occlusions in patients.

### 3.5. Biomarker Levels in Patients with MCMI (≤4 mm)+OVCO, MSCI (<15 mm)+TVCO, and MLCI (≥15 mm)+MVCO

The levels of MDA, ROS, TBARS, and AOPP were increased in MSCI (<15 mm)+TVCO group when compared with CON and MCMI (≤4 mm)+OVCO groups, respectively (*P* < 0.001), and were further increased in MLCI (≥15 mm)+MVCO group compared to MCMI (≤4 mm)+OVCO and MSCI (<15 mm)+TVCO groups, respectively (*P* < 0.001). The expression levels of SOD 1, SOD 2, SOD 3, and PON 1 were decreased in MSCI (<15 mm)+TVCO group when compared with CON and MCMI (≤4 mm)+OVCO groups, respectively (*P* < 0.001), and were further decreased in MLCI (≥15 mm)+MVCO group compared to MCMI (≤4 mm)+OVCO and MSCI (<15 mm)+TVCO groups, respectively (*P* < 0.001). The results suggested a correlation between increased prooxidative stress markers and MCMI+OVCO, MSCI+TVCO, and MLCI+MVCO and also indicated that decreased levels of antioxidative stress markers were linked to severity of MCI+coronary chronic total occlusions in patients (Table 5).

### 3.6. Incidences of MCI+Coronary Occlusions in Elderly Patients

The incidences of MCI+MCTCO in 66-76 and 77-87 years old age groups was higher than 55-65 years old age group. These results showed that the MCI and MCTCO are more frequent in 66-76 and 77-87 years old age groups than in 55-65 years old age group (Table 6).

### 3.7. Multiple Regression Analysis to Assess the Statistical Significance of Variables for MCI+MCTCO in Elderly Patients

By multiple regression analysis, MDA, ROS, TBARS, AOPP, SOD 1, SOD 2, SOD 3, and PON 1 were found to be independent risk indicators for the MCI+MCTCO after adjustment for gender, age, CAD, TIA, FHOS, HT, DM, PVD, smoking, AC, MI, AP, and DOI in elderly patients. All *P* values of less than 0.05 were considered to be statistically significant (Table 7).

### 3.8. Multiple Correlative Analysis to Estimate Relationships between the Markers and MCMI+OVCO, MSCI+TVCO, and MLCI+MVCO

Among all measured biomarkers, we found that the antioxidant/prooxidant effects were correlated with MCMI+OVCO, MSCI+TVCO, and MLCI+MVCO (Table 8).

## 4. Discussion

This study highlights the existence of MCI in combination with MCTCO in elderly patients aged 66 to 87 years. We calculated the incidence and risk factors of MCI in combination with MCTCO due to a lack of robust data on MCI in combination with MCTCO in older patients. In our study, imbalance of prooxidant/antioxidant status is an independent risk factor for MCI in combination with MCTCO, and the MCI+ MCTCO occurs most frequently in elderly patients aged 66 to 87 years, further leading to cerebral stroke and acute myocardial infarction. The significances of clinical findings of this study in evaluating the prooxidative/antioxidative status of MCI and MCTCO in older patients lie in prooxidative and antioxidative markers which were related to the occurrence, development, and prognosis of MCI and MCTCO in older patients, and the markers can be potential targets for diagnosis and treatment of MCI and MCTCO in older patients. Evaluations of prooxidant/antioxidant markers may also be a useful tool for risk assessment and risk stratification of MCI in combination with MCTCO to prevent cerebral stroke and acute myocardial infarction in the older patients. These data may assist clinicians to make informed decisions about early predicting and preventing MCI in combination with MCTCO in older patients before they manifest.

In the present study, it was found that the prooxidative stress markers (MDA, ROS, TBARS, and AOPP) were significantly increased in elderly patients with MCI+MCTCO, indicating that there was a significant relationship between prooxidative stress and MCI+MCTCO in elderly patients. The results showed that the prooxidative stress markers had the predictive values in the prognostic evaluation of elderly patients with concomitant MCI and MCTCO. Oxidative stress leads to ROS overproduction and plays a key role in the pathogenesis of brain ischemia/reperfusion injury. Oxidative damage increases cerebral hemorrhagic infarct and infarct size [34]. Oxidative stress is related to atherosclerosis and increased in patients with acute coronary syndrome and promotes the development of CAD [35]. MDA promotes cerebral ischemia-reperfusion injury through oxidative stress response [36]. MDA-modified low-density lipoprotein is associated with an atherogenic marker and is involved in myocardial infarction [37]. ROS is the major mechanism involving in the procession of cerebral ischemic injury and plays a key role in brain ischemic injury [38]. The intracellular and extracellular ROS is related to atherosclerotic lesions. The high levels of ROS are important in initiation of atherosclerotic lesions and promote advanced atherosclerotic lesion formation [39]. TBARS as a lipid peroxidation marker leads to bilateral carotid artery occlusions and increases cerebral infarct size [40]. Total antioxidant capacity is decreased in the acute myocardial infarction patients by elevating TBARS. TBARS is related to the extent of coronary lesions and promotes development of acute myocardial infarction [41]. AOPP is the marker of oxidant-mediated injury and is associated with carotid atherogenesis and cerebral infarction. AOPP also is a marker of coronary atherosclerosis and plays a key role in CAD [42, 43].

This study also aimed to investigate the relationship between antioxidative markers (SOD 1, SOD 2, SOD 3, and PON 1) and MCI+MCTCO. The results showed that SOD 1, SOD 2, SOD 3, and PON 1 were decreased, and the reduced antioxidative capabilities also were the independent risk factors for MCI+MCTCO in elderly patients. Oxidative stress is an important pathological mechanism of cerebral infarction. High levels of oxidative stress inhibit the antioxidant capacity of SODs and lead to the imbalance between prooxidative stress and antioxidative stress and further promote oxidative damage to the cerebral tissue [44]. Oxidative stress induces the severe pathological changes in the heart tissue and can damage the myocardium. The myocardial activities of SODs as the antioxidants reduce oxidative stress response and suppress the progression of CAD [45]. PON 1 activity as an antiatherogenic factor prevents oxidative modification of low-density lipoprotein and plays an important role in inhibiting the development of cerebral atherosclerosis. PON 1 activity is decreased in patients with carotid atherosclerosis and cerebral atherosclerosis, and a decreased level of PON 1 is associated with cerebral stroke [46]. PON 1 also is the antioxidant enzyme and antiatherosclerotic element located in high-density lipoprotein, and a decreased PON 1 is related to severity and extent of CAD [47].

We demonstrated that the high levels of prooxidative markers induce oxidative stress by inhibiting antioxidative markers. The antioxidative markers are the first line of inhibiting prooxidant enzymes and scavenging oxidative stress activities, and the increase oxidative stress impaired antioxidant defences and showed increased susceptibility to severe cerebral and coronary atherosclerosis. High level of oxidative stress was related to prooxidative and antioxidative imbalance and was involved in the pathogenesis of MCI+MCTCO. Thus, prooxidative and antioxidative imbalance may be one of the important molecular mechanisms contributing to MCI process and accompanying MCTCO in elderly patients. An interplay between prooxidative and antioxidative stress maintains the physiologic level of ROS by antioxidative stress enzymes and high level of oxidative stress is considered as prooxidative and antioxidative imbalance [48]. MCI leads to severer neurological deficits, larger brain infarct sizes, and severer ischemic stroke [49]. MCI occurs in multiple cerebral vascular territories and has a high-risk of cardiac embolism and large arterial atherosclerosis. MCI is an independent risk factor for all-cause mortality [50, 51]. MCTCO is high surgical risk of coronary occlusions and leads to angina pectoris, myocardial infraction, and cardiac shock and strongly is associated with elevated risks of cardiac death and major adverse cardiac and cerebral events [52–55]. Therefore, oxidant/antioxidant imbalance can promote MCI and MCTCO and leads to major adverse cardiovascular and cerebral events. It may be used as the clinical predictive markers for MCI+MCTCO in elderly patients and this study may play an important role in clinical geriatric researches.

Our study further indicated that prooxidative stress markers (MDA, ROS, TBARS, and AOPP) were upregulated and antioxidative markers (SOD 1, SOD 2, SOD 3, and PON 1) were downregulated, suggesting that the antioxidant defence system was likely damaged as indicated by the significantly lower levels of antioxidative markers (SOD 1, SOD 2, SOD 3, and PON 1), and prooxidative and antioxidative imbalance may be involved in the pathogenesis and development of MCI+MCTCO in elderly patients. The changes in the oxidative stress/antioxidant status could suggest mechanisms underlying the development of MCI+MCTCO in elderly patients.

Atherosclerosis is the most common causes of both cerebral artery and coronary artery steno-occlusion. Clinically, cerebral artery and coronary artery steno-occlusion are manifested as a cerebral stroke and acute myocardial infarction [56, 57]. Under physiological conditions, there is an interaction between the level of ROS and the antioxidant defence enzyme expression [57, 58]. The imbalance of oxidative stress resulting from high-level ROS and antioxidation system leads to artery endothelial dysfunction, which is a major determinant of atherosclerosis. Increased oxidative stress contributes to an accumulation of oxidized low-density lipoprotein (OX-LDL), which plays an important role in steno-occlusion of cerebral and coronary arteries [59]. Oxidative stress through ROS promotes artery fatty streaks in atherosclerosis lesions containing foam cells. Artery fatty streaks are subendothelial accumulation of lipid-laden macrophages and are the first step in atherosclerosis in arteries. The activation of artery endothelial cells promotes the differentiation of monocytes into macrophages to oxidize lipids (OX-LDL) and subsequently arterial foam cell formation involving the progression of steno-occlusion of artery atherosclerosis [59]. Therefore, under pathological conditions (atherosclerotic steno-occlusion of arteries), both prooxidant and antioxidant enzyme activities are modified by excessive ROS production. The reduction of antioxidant proteins, elevation of OX-LDL uptake, and downregulation of cholesterol efflux transporters have proatherogenic effects and an increased susceptibility to atherosclerotic steno-occlusion of arteries [59]. The decreased antioxidant enzymes are not capable to inhibit the activity of prooxidant enzymes and eliminate oxidative injury and thus reduced expressions of antioxidant enzymes are responsible for accelerating the progression of cerebral artery and coronary steno-occlusion [60]. Atherosclerosis is increasingly also regarded as a chronic inflammatory process in the arteries and is characterized by lipid accumulation and proinflammatory cell deposits in the walls of cerebral and coronary arteries [58]. Atherosclerotic lesions in the artery walls contribute to the proinflammatory microenvironment of the atherosclerotic progression. Proinflammatory response may also occur as a consequence of oxidative stress response due to elevated ROS [59].

We studied the mechanisms of multiple cerebral infarctions and multiple coronary total occlusions in elderly patients. At least two distinct mechanisms were closely linked to development of cerebral and coronary artery steno-occlusion. First, our research identified that the biomarkers of prooxidant/antioxidant imbalance caused oxidative stress (elevated levels of prooxidant markers including MDA, ROS, TBARS, and AOPP and decreased levels of antioxidant biomarkers including SOD 1, SOD 2, SOD 3, and PON 1) and the unbalanced prooxidant and antioxidant status were an important factor in the development of cerebral and coronary artery steno-occlusion. Our results suggested that the increased prooxidant status (MDA, ROS, TBARS, and AOPP) contributed to the differentiation of monocytes into macrophages to oxidize lipids (OX-LDL) in atherosclerosis lesions of the arterial walls and subsequently arterial foam cell formation involving the progression of cerebral and coronary artery steno-occlusion through inhibiting the expressions of antioxidant enzymes (SOD 1, SOD 2, SOD 3, and PON 1). Second, the artery endothelial dysfunction through proinflammatory response is also considered to be an important event associated to atherogenesis [60]. In this research, oxidative stress possibly occurred in parallel with accelerated onset of proinflammatory signaling pathways and overexpressions of proinflammatory cytokines. Activation of proinflammatory signaling pathways resulted in the infiltration of proinflammatory cells into the artery walls [60] and also played a key role in progressions of cerebral and coronary artery steno-occlusion in patients.

## 5. Conclusion

Prooxidant markers were increased and antioxidant markers were decreased in elderly patients with MCI combined with MCTCO, suggesting that the imbalance between prooxidant/antioxidant status may be involved in the pathogenesis of concomitant MCI and MCTCO in elderly patients.

## Figures and Tables

**Table 1:** The characteristics of patients with MCI+OVCL, MCI+TVCL, and MCI+MVCL.

	CON *n* = 61	MCI+OVCL *n* = 230	MCI+TVCL *n* = 222	MCI+MVCL *n* = 213	*P* ^∗^ values
Gender					
Male, *n* (%)	30 (49)	113 (49)	107 (48)	108 (51)	0.99
Female, *n* (%)	31 (51)	117 (51)	115 (52)	105 (49)	0.10
Age (years)	68.3 ± 11.2	67.6 ± 12.5	69.4 ± 13.0	72.1 ± 14.2	0.97
CAD, *n* (%)	0	230 (100)	222 (100)	213 (100)	1.04
TIA, *n* (%)	0	69 (30)	68 (31)	68 (32)	0.85
FHOS, *n* (%)	0	22 (9)	24 (11)	26 (12)	0.69
HT, *n* (%)	0	34 (15)	36 (16)	36 (17)	0.93
DM, *n* (%)	0	25 (11)	27 (12)	26 (12)	0.87
PVD, *n* (%)	0	11 (5)	16 (7)	17 (8)	0.70
Smoking, *n* (%)	0	7 (3)	9 (4)	9 (4)	0.91
AC, *n* (%)	0	6 (3)	7 (3)	9 (4)	0.90
MI, *n* (%)	0	6 (3)	22 (10)	21 (10)	0.11
AP, *n* (%)	0	7 (3)	31 (14)	30 (15)	0.08
DOI (years), *n* (%)	0	13 (6)	17 (8)	19 (9)	0.73

^∗^Significance via chi-square test. MCI: multiple cerebral infarcts; OVCL: one-vessel coronary lesion; TVCL: two-vessel coronary lesion; MVCL: multiple-vessel coronary lesion; CAD: coronary artery disease; TIA: transient ischemic attack; FHOS: family histories of stroke; HT: hypertension; DM: diabetes mellitus; PVD: peripheral vascular disease; AC: alcohol consumption; MI: myocardial infarction; AP: angina pectoris; DOI: duration of illness.

**Table 2:** Marker levels of oxidative stress in patients with brain infarcts and coronary occlusions.

	CON *n* = 61	MCI+OVCO *n* = 59	MCI+TVCO *n* = 57	MCI+MVCO *n* = 54
MDA (nmol/L)	1.5 ± 0.2	2.1 ± 0.4^∗^	2.9 ± 0.6^∗∗^	3.7 ± 0.7^∗∗∗^
ROS (MFI)	24.6 ± 3.7	35.8 ± 7.8^∗^	46.3 ± 9.4^∗∗^	64.0 ± 12.1^∗∗∗^
TBARS (*μ*mol/L)	2.9 ± 0.4	3.7 ± 0.7^∗^	4.9 ± 0.9^∗∗^	6.0 ± 1.3^∗∗∗^
AOPP (*μ*mol/L)	0.9 ± 0.1	1.9 ± 0.3^∗^	2.7 ± 0.5^∗∗^	4.1 ± 0.8^∗∗∗^
SOD 1 (ng/mL)	25.4 ± 4.1	15.3 ± 2.9^∗^	10.0 ± 2.1^∗∗^	2.8 ± 1.3^∗∗∗^
SOD 2 (ng/mL)	32.7 ± 5.3	23.0 ± 3.5^∗^	9.2 ± 2.0^∗∗^	2.6 ± 1.4^∗∗∗^
SOD 3 (ng/mL)	38.2 ± 6.5	27.1 ± 4.2^∗^	9.3 ± 3.5^∗∗^	2.7 ± 0.7^∗∗∗^
PON 1 (U/L)	151.0 ± 29.5	140.0 ± 27.5^∗^	126.1 ± 25.4^∗∗^	115.8 ± 23.0^∗∗∗^

^∗^ *P* < 0.001 (CON group/MCI+OVCO group). ^∗∗^*P* < 0.001 (MCI+OVCO group/MCI+TVCO group). ^∗∗∗^*P* < 0.001 (MCI+TVCO group/MCI+MVCO group). Group comparisons (CON group/MCI+OVCO group/MCI+TVCO group/MCI+MVCO group) were made using ANOVA, *P* < 0.001. MCI: multiple cerebral infarcts; OVCO: one-vessel coronary occlusion; TVCO: two-vessel coronary occlusion; MVCO: multiple-vessel coronary occlusion; MDA: malondialdehyde; ROS: reactive oxygen species; TBARS: thiobarbituric acid reactive substance; AOPP: advanced oxidation protein products; SOD 1: superoxide dismutase 1; SOD 2: superoxide dismutase 2; SOD 3: superoxide dismutase 3; PON 1: paraoxonase 1.

**Table 3:** Levels of biomarkers in patients with UMCI+OVCO, BMCI+TVCO, and BMCI+MVCO.

	CON *n* = 61	UMCI+OVCO *n* = 57	BMCI+TVCO *n* = 55	BMCI+MVCO *n* = 53
MDA (nmol/L)	1.5 ± 0.2	2.6 ± 0.5^∗^	3.7 ± 0.8^∗∗^	5.5 ± 1.3^∗∗∗^
ROS (MFI)	24.6 ± 3.7	40.1 ± 7.1^∗^	52.0 ± 9.6^∗∗^	73.4 ± 12.6^∗∗∗^
TBARS (*μ*mol/L)	2.9 ± 0.4	4.1 ± 0.8^∗^	5.9 ± 1.1^∗∗^	7.1 ± 1.4^∗∗∗^
AOPP (*μ*mol/L)	0.9 ± 0.1	2.1 ± 0.4^∗^	4.4 ± 0.8^∗∗^	5.9 ± 1.4^∗∗∗^
SOD 1 (U/mL)	25.4 ± 4.1	16.2 ± 3.2^∗^	7.2 ± 1.4^∗∗^	2.0 ± 0.5^∗∗∗^
SOD 2 (U/mL)	32.7 ± 5.3	23.5 ± 4.0^∗^	12.2 ± 2.4^∗∗^	2.1 ± 0.3^∗∗∗^
SOD 3 (U/mL)	38.2 ± 6.5	29.9 ± 5.2^∗^	13.1 ± 2.6^∗∗^	1.9 ± 0.4^∗∗∗^
PON 1 (U/L)	151.0 ± 29.5	140.0 ± 27.9^∗^	132.5 ± 26.1^∗∗^	120.1 ± 24.5^∗∗∗^

^∗^ *P* < 0.001 (CON group/UMCI+OVCO group). ^∗∗^*P* < 0.001 (UMCI+OVCO group/BMCI+TVCO group). ^∗∗∗^*P* < 0.001 (BMCI+TVCO group/BMCI+MVCO group). Group comparisons (CON group/UMCI+OVCO group/BMCI+TVCO group/BMCI+MVCO group) were made using ANOVA, *P* < 0.001. UMCI: unilateral multiple cerebral infarcts; OVCO: one-vessel coronary occlusion; BMCI: bilateral multiple cerebral infarcts; TVCO: two-vessel coronary occlusion; MVCO: multiple-vessel coronary occlusion; MDA: malondialdehyde; ROS: reactive oxygen species; TBARS: thiobarbituric acid reactive substance; AOPP: advanced oxidation protein products; SOD 1: superoxide dismutase 1; SOD 2: superoxide dismutase 2; SOD 3: superoxide dismutase 3; PON 1: paraoxonase 1.

**Table 4:** Changes of biomarkers in patients with MLCI+OVCO, MCBI+TVCO, and MCBI+MVCO.

	CON *n* = 61	MLCI+OVCO *n* = 57	MCBI+TVCO *n* = 55	MCBI+MVCO *n* = 53
MDA (nmol/L)	1.5 ± 0.2	2.9 ± 0.5^∗^	4.0 ± 0.7^∗∗^	6.1 ± 1.2^∗∗∗^
ROS (MFI)	24.6 ± 3.7	37.0 ± 7.1^∗^	48.8 ± 9.0^∗∗^	59.3 ± 13.4^∗∗∗^
TBARS (*μ*mol/L)	2.9 ± 0.4	4.5 ± 0.8^∗^	6.7 ± 1.3^∗∗^	8.9 ± 1.8^∗∗∗^
AOPP (*μ*mol/L)	0.9 ± 0.1	2.1 ± 0.4^∗^	3.0 ± 0.6^∗∗^	4.7 ± 0.9^∗∗∗^
SOD 1 (U/mL)	25.4 ± 4.1	16.3 ± 3.0^∗^	6.7 ± 1.4^∗∗^	1.1 ± 0.2^∗∗∗^
SOD 2 (U/mL)	32.7 ± 5.3	21.3 ± 4.6^∗^	12.8 ± 2.0^∗∗^	2.9 ± 0.4^∗∗∗^
SOD 3 (U/mL)	38.2 ± 6.5	26.8 ± 5.1^∗^	14.5 ± 2.7^∗∗^	3.6 ± 0.7^∗∗∗^
PON 1 (U/L)	151.0 ± 29.5	142.7 ± 28.3^∗^	130.1 ± 25.0^∗∗^	119.9 ± 22.8^∗∗∗^

^∗^ *P* < 0.001 (CON group/MLCI+OVCO group). ^∗∗^*P* < 0.001 (MLCI+OVCO group/MCBI+TVCO group). ^∗∗∗^*P* < 0.001 (MCBI+TVCO group/MCBI+MVCO group). Group comparisons (CON group/MLCI+OVCO group/MCBI+TVCO group/MCBI+MVCO group) were made using ANOVA, *P* < 0.001. MLCI: multiple lacunar cerebral infarcts; OVCO: one-vessel coronary occlusion; MCBI: multiple cerebellum infarcts; TVCO: two-vessel coronary occlusion; MVCO: multiple-vessel coronary occlusion; MDA: malondialdehyde; ROS: reactive oxygen species; TBARS: thiobarbituric acid reactive substance; AOPP: advanced oxidation protein products; SOD 1: superoxide dismutase 1; SOD 2: superoxide dismutase 2; SOD 3: superoxide dismutase 3; PON 1: paraoxonase 1.

**Table 5:** Levels of biomarkers in patients with MCMI+OVCO, MSCI+TVCO, and MLCI+MVCO.

	CON *n* = 61	MCMI (≤4 mm)+OVCO *n* = 57	MSCI (<15 mm)+TVCO *n* = 55	MLCI (≥15 mm)+MVCO *n* = 53
MDA (nmol/L)	1.5 ± 0.2	2.7 ± 0.5^∗^	4.6 ± 0.9^∗∗^	6.9 ± 1.4^∗∗∗^
ROS (MFI)	24.6 ± 3.7	39.0 ± 7.5^∗^	50.1 ± 9.8^∗∗^	71.8 ± 13.6^∗∗∗^
TBARS (*μ*mol/L)	2.9 ± 0.4	4.0 ± 0.8^∗^	5.2 ± 1.0^∗∗^	7.0 ± 1.4^∗∗∗^
AOPP (*μ*mol/L)	0.9 ± 0.1	1.9 ± 0.3^∗^	2.9 ± 0.5^∗∗^	4.3 ± 0.8^∗∗∗^
SOD 1 (U/mL)	25.4 ± 4.1	17.0 ± 2.0^∗^	7.6 ± 1.3^∗∗^	1.9 ± 0.2^∗∗∗^
SOD 2 (U/mL)	32.7 ± 5.3	23.9 ± 4.6^∗^	13.8 ± 2.8^∗∗^	2.7 ± 0.5^∗∗∗^
SOD 3 (U/mL)	38.2 ± 6.5	26.7 ± 5.0^∗^	14.6 ± 2.9^∗∗^	4.0 ± 0.8^∗∗∗^
PON 1 (U/L)	151.0 ± 29.5	139.1 ± 28.9^∗^	121.9 ± 24.38^∗∗^	110.0 ± 22.0^∗∗∗^

^∗^ *P* < 0.001 (CON group/MCMI (≤4 mm)+OVCO group). ^∗∗^*P* < 0.001 (MCMI (≤4 mm)+OVCO/MSCI (<15 mm)+TVCO group). ^∗∗∗^*P* < 0.001 (MSCI (<15 mm)+TVCO group/MLCI (≥15 mm)+MVCO group). Group comparisons (CON group/MCMI (≤4 mm)+OVCO group/MSCI (<15 mm)+TVCO group/MLCI (≥15 mm)+MVCO group) were made using ANOVA, *P* < 0.001. MCMI: multiple cerebral microinfarcts; OVCO: one-vessel coronary occlusion; MSCI: multiple small cerebral infarcts; TVCO: two-vessel coronary occlusion; MLCI: multiple lacunar cerebral infarcts; MVCO: multiple-vessel coronary occlusion; MDA: malondialdehyde; ROS: reactive oxygen species; TBARS: thiobarbituric acid reactive substance; AOPP: advanced oxidation protein products; SOD 1: superoxide dismutase 1; SOD 2: superoxide dismutase 2; SOD 3: superoxide dismutase 3; PON 1: paraoxonase 1.

**Table 6:** The incidences of MCI+MCTCO in different groups.

Age groups (years)	MCI+OVCO *n* = 59	MCI+TVCO *n* = 57	MCI+MVCO *n* = 54
55-65, *n* (%)	30 (50)	14 (24)	9 (17)
66-76, *n* (%)	20 (34)^∗^	19 (34)^∗^	18 (34)^∗^
77-87, *n* (%)	9 (15)^∗∗^	24 (42)^∗∗^	27 (50)^∗∗^

Age groups (years)	UMCI+OVCO *n* = 57	BMCI+TVCO *n* = 55	BMCI+MVCO *n* = 53
55-65, *n* (%)	25 (44)	13 (24)	12 (23)
66-76, *n* (%)	19 (34)^∗^	19 (34)^∗^	18 (34)^∗^
77-87, *n* (%)	13 (22)^∗∗^	23 (42)^∗∗^	23 (43)^∗∗^

Age groups (years)	MLCI+OVCO *n* = 57	MCBI+TVCO *n* = 55	MCBI+MVCO *n* = 53
55-65, *n* (%)	30 (52)	9 (17)	11 (2)
66-76, *n* (%)	20 (35)^∗^	19 (34)^∗^	19 (36)^∗^
77-87, *n* (%)	7 (13)^∗∗^	27 (49)^∗∗^	33 (62)^∗∗^

Age groups (years)	MCMI (≤4 mm)+OVCO *n* = 57	MSCI (<15 mm)+TVCO *n* = 55	MLCI (≥15 mm)+MVCO *n* = 53
55-65, *n* (%)	28 (49)	10 (19)	7 (13)
66-76, *n* (%)	18 (32)^∗^	18 (32)^∗^	18 (34)^∗^
77-87, *n* (%)	11 (19)^∗∗^	27 (49)^∗∗^	28 (52)^∗∗^

Significance via chi-square test. ^∗^*P* < 0.05 (55-65 age group/66-76 age group). ^∗∗^*P* < 0.05 (66-76 age group/77-87 age group). Group comparisons (55-65 age group/66-76 age group/77-87 age group) were made using ANOVA, *P* < 0.05. MCI: multiple cerebral infarcts; MCTCO: multiple chronic total coronary occlusions; OVCO: one-vessel coronary occlusion; TVCO: two-vessel coronary occlusion; MVCO: multiple-vessel coronary occlusion; MLCI: multiple lacunar cerebral infarcts; BMCI: bilateral multiple cerebral infarcts; MLCI: multiple lacunar cerebral infarcts; MCBI: multiple cerebellum infarcts; MCMI: multiple cerebral microinfarcts; MSCI: multiple small cerebral infarcts; MLCI: multiple large cerebral infarcts.

**Table 7:** Multiple regression analysis of risk indicators for MCI+MCTCO.

Variables	Odds ratio	95% CI	*P* value
Gender	2.19	0.30-8.11	0.35
Age	1.25	0.26-10.30	0.21
CAD, *n* (%)	1.34	0.25-9.01	0.16
TIA, *n* (%)	2.19	0.30-11.43	0.30
FHOS, *n* (%)	4.05	0.21-10.26	0.45
HT, *n* (%)	1.27	0.48-9.02	0.31
DM, *n* (%)	2.40	0.35-10.11	0.45
PVD, *n* (%)	1.36	0.20-13.30	0.19
Smoking, *n* (%)	3.24	0.52-8.63	0.50
AC, *n* (%)	1.50	0.28-10.14	0.17
MI, *n* (%)	2.48	0.50-14.39	0.28
AP, *n* (%)	4.27	0.53-16.14	0.16
DOI (years), *n* (%)	1.15	0.29-13.07	0.35
MDA	4.20	1.30-17.69	0.001
ROS	5.17	1.46-19.02	0.001
TBARS	3.45	1.31-3.70	0.02
AOPP	3.12	1.40-2.68	0.04
SOD 1	2.58	1.37-4.31	0.01
SOD 2	4.31	1.52-2.87	0.02
SOD 3	5.79	1.64-13.45	0.001
PON 1	4.26	1.47-18.12	0.001

MCI: multiple cerebral infarcts; MCTCO: multiple coronary total chronic occlusions; CAD: coronary artery disease; TIA: transient ischemic attack; FHOS: family histories of stroke; HT: hypertension; DM: diabetes mellitus; PVD: peripheral vascular disease; AC: alcohol consumption; MI: myocardial infarction; AP: angina pectoris; DOI: duration of illness; MDA: malondialdehyde; ROS: reactive oxygen species; TBARS: thiobarbituric acid reactive substance; AOPP: advanced oxidation protein products; SOD 1: superoxide dismutase 1; SOD 2: superoxide dismutase 2; SOD 3: superoxide dismutase 3; PON 1: paraoxonase 1.

**Table 8:** Relationships between biomarkers and MCMI+OVCO, MSCI+TVCO, and MLCI+MVCO.

	CON		MCMI+OVCO		MSCI+TVCO		MLCI+MVCO	
	*r*	*P*	*r*	*P*	*r*	*P*	*r*	*P*
MDA	-0.07	0.43	0.46	0.03	0.81	0.001	0.79	0.002
ROS	0.05	0.25	0.53	0.02	0.77	0.02	0.56	0.03
TBARS	-0.04	0.07	0.61	0.03	0.46	0.03	0.61	0.01
AOPP	-0.06	0.18	0.56	0.01	0.69	0.02	0.45	0.03
SOD 1	-0.07	0.35	0.80	0.001	0.92	0.001	0.82	0.01
SOD 2	-0.02	0.81	0.56	0.01	0.80	0.002	0.73	0.003
SOD 3	-0.04	0.09	0.41	0.04	0.56	0.03	0.47	0.04
PON 1	0.08	0.60	0.74	0.001	0.86	0.002	0.92	0.001

MCMI: multiple cerebral microinfarcts; OVCO: one-vessel coronary occlusion; MSCI: multiple small cerebral infarcts; TVCO: two-vessel coronary occlusion; MLCI: multiple large cerebral infarcts; MVCO: multiple-vessel coronary occlusion; MDA: malondialdehyde; ROS: reactive oxygen species; TBARS: thiobarbituric acid reactive substance; AOPP: advanced oxidation protein products; SOD 1: superoxide dismutase 1; SOD 2: superoxide dismutase 2; SOD 3: superoxide dismutase 3; PON 1: paraoxonase 1.

## Data Availability

All relevant data are within this research paper. All data used to support the findings of the research are available from the corresponding author on reasonable. No additional data are available.
